# A whey protein supplement decreases post-prandial glycemia

**DOI:** 10.1186/1475-2891-8-47

**Published:** 2009-10-16

**Authors:** Brent L Petersen, Loren S Ward, Eric D Bastian, Alexandra L Jenkins, Janice Campbell, Vladimir Vuksan

**Affiliations:** 1 Glycemic Index Laboratories Inc, Toronto, Ontario, Canada; 2 Glanbia Research and Development Center, Twin Falls, ID, USA

## Abstract

**Background:**

Incidence of diabetes, obesity and insulin resistance are associated with high glycemic load diets. Identifying food components that decrease post-prandial glycemia may be beneficial for developing low glycemic foods and supplements. This study explores the glycemic impact of adding escalating doses of a glycemic index lowering peptide fraction (GILP) from whey to a glucose drink.

**Methods:**

Ten healthy subjects (3M, 7F, 44.4 ± 9.3 years, BMI 33.6 ± 4.8 kg/m^2^) participated in an acute randomised controlled study. Zero, 5, 10 and 20 g of protein from GILP were added to a 50 g glucose drink. The control (0 g of GILP) meal was repeated 2 times. Capillary blood samples were taken fasting (0 min) and at 15, 30, 45, 60, 90 and 120 minutes after the start of the meal and analyzed for blood glucose concentration.

**Results:**

Increasing doses of GILP decreased the incremental areas under the curve in a dose dependant manner (Pearson's r = 0.48, p = 0.002). The incremental areas (iAUC) under the glucose curve for the 0, 5, 10, and 20 g of protein from GILP were 231 ± 23, 212 ± 23, 196 ± 23, and 138 ± 13 mmol.min/L respectively. The iAUC of the 20 g GILP was significantly different from control, 5 g GILP and 10 g GILP (p < 0.001). Average reduction in the glucose iAUC was 4.6 ± 1.4 mmol.min/L per gram of ingested GILP.

**Conclusion:**

Addition of GILP to a oral glucose bolus reduces blood glucose iAUC in a dose dependent manner and averages 4.6 ± 1.4 mmol.min/L per gram of GILP. These data are consistent with previous research on the effect of protein on the glycemic response of a meal.

## Background

The rate of glucose absorption and the duration of elevated blood glucose levels induce many hormonal and metabolic changes that may affect health or disease parameters. Low glycemic index (GI) diets may help in weight maintenance and weight loss [1] in addition to being protective against chronic disease such as diabetes [2], heart disease [3,4] and certain cancers [5,6]. Interest in identifying low GI foods and the food factors responsible for the low GI of foods has therefore increased. Several food factors have been identified that influence *in vivo *absorption and therefore potentially the GI of a food or meal. Some of these factors include: the food matrix, cell wall and starch structure (i.e. ripening), amylose to amylopectin ratio, protein-starch interaction, processing and dietary fibre [7].

More recently, interest has focused on the metabolic responses to dietary proteins. Proteins vary in their ability to decrease post-prandial glycemia. Comparing the insulinotropic characteristics of milk, gluten, cod, cheese and whey demonstrated that both milk and whey have the greatest impact on glucose metabolism by increasing both insulin secretion and glucose-dependent insulinotropic polypeptide (GIP) [8].

Milk protein, in particular, appears to stimulate an increase in postprandial insulin response with a corresponding reduction in postprandial blood glucose levels [9,10]. Studies exploring the insulinotropic effect of the dairy protein have found that the whey fraction seems to contain the predominating insulinotropic secretagogue [8,11,12].

Different sources of protein may be digested at different rates; with whey being one of the most rapidly digested resulting in high postprandial concentrations of amino acids [9]. Individual amino acids may act as potent insulin secretagogues [13,14] and in particular, leucine, isoleucine, valine, lysine and threonine have been proposed as the most likely amino acids responsible for the increase seen in insulin concentrations [8]. It is not known if addition of whey protein will consistently lower postprandial blood glucose responses and whether this lowering happens in a dose responsive manner.

This study was therefore conducted to measure the impact of a glycemic index lowering peptide ingredient given in escalating doses to a glucose drink on post prandial glycemia and to calculate the relative glycemic lowering ability of this protein fraction.

## Methods

### Subjects

Ten healthy subjects participated in the study consisting of 3 males and 7 females with an average age of (Mean ± SD) 44.4 ± 9.3 years. The body mass index of the subjects ranged from 26.7 to 41.1 kg/m^2^, the average being 33.6 ± 4.8 kg/m^2^. Subjects were recruited through the Glycemic Index Laboratories clinic volunteer roster. The study was approved by the Western Institutional Review Board, Washington. Informed written consent was obtained from all subjects prior to the start of the study. Subjects received a financial reward for their participation. All 6 treatments were completed on separate days with at least one day between tests.

### Test Meals

The test meals (Table 1) consisted of 250 ml of water blended with 50 g of anhydrous glucose (control), 50 g of glucose + 5.6 g GILP powder (5 g GILP protein), 50 g of glucose + 11.2 g GILP powder (10 g GILP protein) and 50 g of glucose + 22.5 g GILP powder (20 g GILP protein). Testing of the various meals was done in a randomized fashion. The control glucose meal was repeated 2 times by each subject and the average was calculated.

**Table 1:** Composition of the different meals.

**Component**	**Control**	**5 g GILP**	**10 g GILP**	**20 g GILP**
**Protein (g)**	0	5	10	20
Leucine (g)	0	0.56	1.12	2.24
Isoleucine (g)	0	0.36	0.72	1.44
Valine (g)	0	0.32	0.63	1.26
**Total BCAA**	0	1.24	2.47	4.94
Lysine	0	0.48	0.96	1.92
Cysteine	0	0.13	0.25	0.5
Methionine	0	0.10	0.21	0.42
Tryptophan	0	0.11	0.22	0.44
Phenylalanine	0	0.16	0.32	0.64
Histidine	0	0.09	0.19	0.38
Threonine	0	0.40	0.80	1.60
Arginine	0	0.08	0.16	0.32
Tyrosine	0	0.16	0.31	0.62
**Lipid (g)**	0	0.03	0.06	0.12
**Carbohydrate (g)**	50	50	50	50
Lactose (g)	0	0.07	0.14	0.28
**Minerals**				
Calcium (mg)	0	121	241	482
Phosphorus (mg)	0	63	127	254
Sodium (mg)	0	53	107	214
Potassium (mg)	0	25	50	100
Magnesium (mg)	0	13	25	50
Zinc (ug)	0	15	30	60

### Protein Supplement

The protein supplement (Glanbia, Twin Falls, ID USA) consisted of a blend of whey peptides and intact whey protein containing a high concentration of branch chain amino acids. Doses were calculated to contain 5, 10 and 20 g of protein.

### Protocol

On each test day, subjects came to Glycemic Index Laboratories, Inc. in the morning after a 10-14 h overnight fast. After being weighed and having a fasting blood sample obtained by finger-prick, the subject then consumed a test meal within 10 minutes, and further blood samples were obtained at 15, 30, 45, 60, 90 and 120 minutes after the start of the test meal.

A beverage of choice was served to each subject with each test meal, comprising of one or two cups of water, tea or coffee with or without milk. The same beverage was served to the subject at each of the subsequent test meals.

### Data Analysis

The incremental area under the plasma glucose response curve (iAUC) for each of the test meals for each subject was calculated using the trapezoid rule and not using the area under the baseline [15]. The mean of the two glucose meals (control) was used in statistical analysis and the intra-individual coefficient of variation and intraclass coefficient of correlation of the two OGTTs were calculated. Differences between the meals were assessed using repeated-measures analysis of variance (ANOVA) and adjusted for multiple pair-wise comparisons with the Tukey-Kramer procedure. Relationship between glucose iAUC levels and GILP dose was calculated using the Pearson's correlation and linear regression to estimate the reduction in iAUC per gram of GILP. All data are expressed as Mean ± SEM.

## Results

The means of the iAUC of the two glucose controls were 237 ± 25 and 225 ± 24 mmol/L·min. The intra-individual coefficient of variation and intra-class coefficient of correlation of the repeated control glucose test meals were 28.3% and 17.9% respectively.

Glucose iAUC levels correlated inversely with GILP doses, indicating that higher doses of GILP resulted in lower blood glucose levels (Pearson's *r *= -0.48, p = 0.002). Linear regression analysis showed significant dose-response decrease in blood glucose iAUC with increasing doses of GILP (slope = -4.6 ± 1.4; t = -3.4; p = 0.002; Y = 235-4.6X; *r*^*2 *^= 0.23).

**Figure 1:**
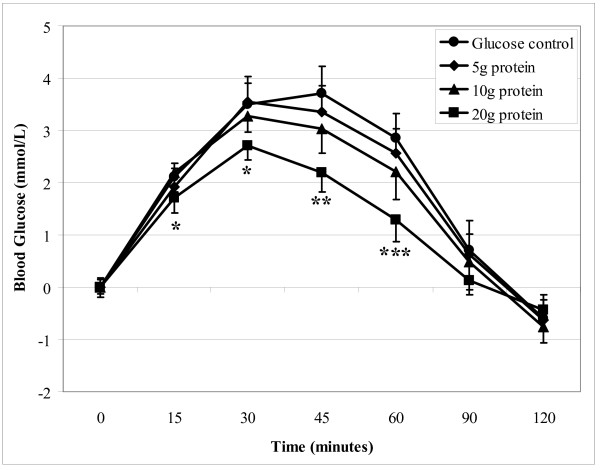
**Incremental blood glucose response after meals containing either glucose alone (control), or glucose plus either 5, 10 or 20 g of protein from GILP**. Data are expressed as Mean ± SEM. * 20 g GILP meal significantly different from control, ** 20 g GILP different from 5 g GILP and control, *** 20 g GILP different from all meals using GLM ANOVA and adjusted for multiple pair-wise comparisons with the Tukey-Kramer test (p < 0.001).

**Figure 2:**
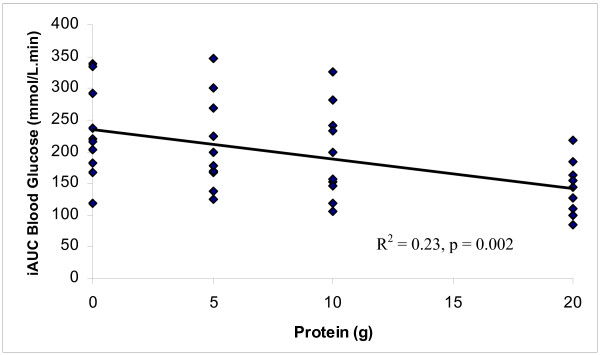
**Correlation of blood glucose iAUC with amount of ingested protein after meals containing either 50 g of glucose alone (0 protein), or 50 g of glucose plus 5, 10 or 20 g of protein from GILP (p = 0.002)**.

Blood glucose concentrations were significantly reduced at 30, 45 and 60 minutes after the 20 g GILP protein meal when compared to control (Figure 1). The blood glucose levels after the 20 g GILP meal were also lower than the 5 g GILP meal at 45 min and both the 5 g and 10 g GILP meals at 60 min (p < 0.001). Incremental areas under the curve (iAUC) for the GILP plus glucose meals of 0, 5, 10 and 20 g of protein were 231 ± 23, 212 ± 23, 196 ± 23, and 138 ± 13 mmol/L·min respectively. The iAUC of 20 g GILP protein plus glucose meal was significantly lower than the glucose control, 5 g GILP protein and 10 g GILP protein meals (p < 0.001) (Figure 2). The 5, 10 and 20 g doses of protein reduced the iAUC by 7.6, 13.3 and 37.5% respectively.

## Discussion

This study demonstrated that addition of this unique glycemic index lowering peptide whey-based protein reduces postprandial glycemia in a dose-dependent manner when added to a 50 g glucose drink. The exact mechanism is not known but it has been postulated that the blood glucose lowering is a result of an increase of insulin secretion [11,12]. In studies where protein is combined with carbohydrate, an enhancement of the insulin response is observed [11,16].

The differential effect of different amino acids on postprandial insulin levels is well known, it is therefore likely that protein mixes with different amino acid profiles will elicit different postprandial insulin responses. Whey protein is particularly high in branch-chain amino acids, in particular leucine [17]. These amino acids are insulinogenic, meaning that they have a higher capacity to increase an insulin response [18], which may account for the results seen in this study. Another possibility is the effect of whey protein on incretin hormones released in the gut [19,20]. Incretin hormones such as Glucagon-like-peptide-1 (GLP-1) and Glucose-dependent insulinotropic peptide (GIP) are released from the gut after food consumption and are involved in many digestive roles including glycemic control.

Glucagon-like peptide-1 (GLP-1) is a multi-functioning hormone which is released from the gut in response to a meal. Exogenous GLP-1 stimulates insulin secretion and individuals with diabetics have lower levels of GLP-1 secretion [21].

Dipeptidyl peptidase IV (DPP-IV) is a peptidase which hydrolyzes incretin hormones like GLP-1. Inhibiting DPP-IV in rats with an inhibitor has been shown to suppress blood glucose elevation by possibly protecting GLP-1 [22]. One important role whey protein may play is in the physiology of incretin hormones. Whey protein has been shown in mice to raise GLP-1 and inhibit DPP-IV resulting in an increased and prolonged insulin response [19,20]. Measurement of these hormones after ingestion of meals containing GILP may help elucidate the mechanism of action of the glucose reduction seen with this whey peptide fraction.

## Conclusion

In conclusion, this unique glycemic index lowering peptide whey based protein significantly lowered the glycemic response elicited by 50 g of glucose in a dose dependent manner. The reduction in blood glucose iAUC per gram of protein powder was calculated to be 4.6 ± 1.4 mmol.min/L. Addition of GILP to carbohydrate food products may be an effective way of lowering the glycemic impact of these foods.

## Competing interests

Glanbia Nutritionals provided financial support for this study. BLP, LSW and EDB are employees of Glanbia Nutritionals. ALJ, VV and JC had no competing interests.

## Authors' contributions

BLP, LSW, EDB, ALJ and VV were involved in the experimental design and manuscript preparation. JC and ALJ were responsible for the running of the clinical trial. ALJ and VV were responsible for the statistical analysis and interpretation of the data.
